# Cases Successfully Treated with Tincture of Iodine

**Published:** 1860-12

**Authors:** E. Nichols

**Affiliations:** Student in the Rush Medical College


### ARTICLE 2.

### CASES SUCCESSFULLY TREATED WITH TINCTURE OF IODINE.

BY E. NICHOLS, STUDENT IN THE RUSH MEDICAL COLLEGE.

I have been led to make the following remarks from the numerous cases of caries I have seen in a short time, since my attention has been directed to it, and for the reason that I have seen no mention made, in any of the works I have read on surgery, of the remedy used successfully in all the cases in which I have seen it tried.

I shall not enter into discussions on the nature of disease; as to whether it is produced by an unhealthy condition of the system, external local injuries, or with no *perceptible cause;* but at once give the case I am most acquainted with, it having occurred in my own person.

At the age of sixteen I was taken with a dull, sometimes darting, aching pain in the region of the os calsis, which pain I attributed to the irritation of a boot crowding painfully upon that region. For a number of days the pain increased, with no swelling of the soft parts, but a slight reddening of the skin. A physician was consulted, and blisters applied to each side of the heel, with no other effect than producing more inflammation, and soon enlargement of the bone took place, and swelling of the soft parts began, which extended from the extremity of the foot to the knee joint. Poultices were now employed, and abscesses formed, which discharged considerable unhealthy pus.

The physician now continuing a course of treatment which consisted of lancing and applying simple cerate, as most patients do under unsuccessful treatment, and at the end of six or eight weeks I placed myself under the care of another physician, who cleared my foot, to some extent, of pus, by lancing and pressure. The proud flesh was taken out by means of scissors and nitrate of silver. Inflammation was removed by the use of acetate of lead and sal ammoniac, of each about half an ounce, dissolved in one pint of cold water, and applied by saturating a cloth and laying it upon the inflamed parts. This was removed as often as the cloth became dry, and was very effectual in removing the inflammation. Citrine ointment was applied over the abscesses, and when the inflammation had somewhat subsided, tincture of iodine was used on the surface in the immediate region of the disease, and inserted into the abscesses. This was applied about once a day, with a camel's hair pencil. When the acute form of the disease had nearly passed, injection of the tincture of iodine were made into the abscesses with a small syringe. These injections were so complete as to pass from an abscess on one side of the heel to an abscess on the opposite side.

Before these injections were made, the bone was examined from time to time with a probe, and though it could be passed in various directions into the substance of the bone, no loose pieces could be detected. But very soon after the injections were made, pieces of bone were found in the abscesses and removed, to the number of twenty, varying in size from a line to an inch in length, and from half a line to three-quarters of an inch in breadth. They were irregular in shape, and often very porous, and appeared to be disorganized. Injections werc continuous till no pieces of loosened bone could be detected, and all bony surfaces touched with the probe seemed firm and healthy, when it was discontinued, and fungous granulations being kept out of the abscesses, they were allowed to heal slowly.

This treatment was commenced in March, and in the ensuing October the disease was perfectly cured, and the foot again became quite serviceable. It is now a good foot, and "keeps pace" with the other, which is and always has been perfectly well.

In this connection I will mention that the dorsal surface of the scaphoid bone was slightly affected, but arrested by a few injections of iodine. Only a few scales of bone were taken from it.

After this there was the appearance of caries on the tibia of the opposite limb, near the insertion of the sartorius mus cle, which, as soon as an abscess was formed, I thoroughly injected with the tincture of iodine, and succeeded in removing a small scale of bone, when it immediately healed. This might have been necrosis; but no scale of bone was detected till after the injection, and the scale seemed disorganized.

During this time all constitutional treatment was in accordance with that laid down in the books.

Since that time I have witnessed similar cases cured by a like treatment; but all happened in short bones. I have not witnessed a trial of this treatment in any long bones. I have also seen cases treated according to rules laid down in the works on surgery, but with altogether less success.

Now, these questions are in my mind: Why is not this treatment adopted? Is is because the profession have not tried it, or has it been tried and proved inadequate? I have not the means of investigating the latter; but have seen no mention of this treatment in any works I have read, and never heard it mentioned by a surgical teacher. Does tincture of iodine produce any bad effect thus applied? I have seen none.

How does the tincture of iodine prove beneficial? Is it by arresting the progress of the disease, as in the case of erysipelas, where the diseased portion is surrounded with it, and does it cause exfoliation of the bone as it does desduamation of the skin?

I have written this because the above are questions I am not able to answer, and I have not the means of investigating the matter as I should desire. If it is an adequate treatment I think it should be adopted, as it less painful, and I think far less dangerous than the various operations usually adopted in the cure of this disease.

---

